# Sex differences in children operated with pyeloplasty for pelvoureteric junction obstruction

**DOI:** 10.1007/s00383-023-05543-6

**Published:** 2023-09-08

**Authors:** Linnea Högberg, Sanni Värelä, Magnus Anderberg, Martin Salö

**Affiliations:** 1 Department of Pediatric Surgery, Skåne University Hospital, Lund, Sweden; 2 Department of Clinical Sciences, Pediatrics, Lund University, Lasarettsgatan 48, 221 85 Lund, Sweden; 3 Department of Surgery, Skåne University Hospital, Malmö, Sweden

**Keywords:** Ureteropelvic obstruction, Sex differences, Pyeloplasty, Pediatric urology, Reoperation

## Abstract

**Purpose:**

Pelvoureteric junction obstruction (UPJO) is a common cause of hydronephrosis in children but no previous studies have evaluated differences between boys and girls operated with pyeloplasty. This study aimed to evaluate potential differences between sexes in children operated with pyeloplasty for PUJO in terms of presentation, surgery, and long-term results.

**Methods:**

Data was retrospectively collected from all children operated on with pyeloplasty between January 2002 and December 2020. Data contained several variables covering presentation, surgery, and long-term results.

**Results:**

In total, 194 patients were included of which 126 (64.9%) were boys. There were no significant differences in prenatal findings, pelvic dilation on ultrasound, function of the affected kidney, surgical method, obstruction type, resolution of hydronephrosis, or improvement of function. Boys presented with pain more often than girls (47.4 vs 25.0%, *p* < 0.01) while girls were more prone to infections preoperatively (17.2 vs 7.0%, *p* = 0.04). All nine patients requiring reoperation were boys (*p* = 0.03).

**Conclusion:**

Girls with UPJO seem to experience infections as presenting symptoms more often than boys, while boys significantly more often present with pain. There is also a higher percentage of boys needing reoperation.

## Introduction

Pelvoureteric junction obstruction (UPJO) is an obstruction of the proximal ureter in the renal hilum causing hydronephrosis [1] and it is the most common cause of prenatal hydronephrosis [1, 2]. The condition is much more common in boys with a ratio between 2 and 3:1 [3–5]. While many cases of UPJO are discovered as prenatal hydronephrosis, others present later in childhood with pain or pyelonephritis [6]. Around 20% of the patients with UPJO will eventually need a pyeloplasty [7, 8]. Pyeloplasty improves long-term renal function [9, 10] and about 90–100% of patients achieve resolution of their hydronephrosis after pyeloplasty [11, 12].

Previous, large American studies in pediatric surgery have found girls to have lower risk of postoperative complications in gastrointestinal surgery [13, 14]. Further, Bhattacharyya et al. found girls to have a reduced risk of postoperative bleeding after tonsillectomy [15]. On the opposite, female sex has been associated with worse postoperative outcomes after pyloromyotomy [16]. There may also be differences when it comes to seeking and receiving health care, though research in the pediatric field is sparse. Zachariasse et al. found that boys were overly represented in European emergency departments while girls received more diagnostic testing [17]. As it seems to exist differences between sexes in other types of pediatric surgeries, it is valuable to know if the disparities are present in UPJO and pyeloplasty as well. There are several suggestions as to why men and women may have different pathophysiology, treatment, and surgery outcomes, including both biological and sociocultural reasons [18].

When it comes to UPJO and pyeloplasty, there is not much research performed on the subject. Previous studies have indicated that girls have a higher rate of crossing vessels [19] and another study found no difference in who needs surgery [5] but to the best of our knowledge, there is no study with the main aim to explore sex differences in UPJO and pyeloplasty. Identifying patient related factors, such as sex, that may affect outcome after surgery is important for future studies, as part of information to patients and family and perhaps also for planning surgery and follow-up. It is also important to notice potential delays or sex difference in how a disease presents.

Thus, this article compares boys and girls in a retrospective cohort from almost 20 years back and aims to evaluate potential differences between boys and girls being operated with pyeloplasty for ureteropelvic junction obstruction in terms of presentation, surgery, and long-term results.

## Material and method

This study was approved by the national ethical authority (DNR no 2021-0480) and by the hospital’s own ethical board (KVB no 2019-19).

### Settings and study population

Patients were treated at a tertiary center performing advanced pediatric surgery with a catchment area of approximately 2 million people. All children < 15 years in this region needing pyeloplasty were referred to the center.

All children who underwent pyeloplasty between January 2002—December 2020 were eligible for inclusion. Indications for pyeloplasty, consistent over the study period were: increase in APD (or very high at presentation), DRF < 40%, pain or increased APD + pathological MAG curve.

Bilaterally operated patients were included in analysis of sex and side of surgery but excluded from all other statistics. Unilaterally operated patients with bilateral hydronephrosis were excluded from pre- and postoperative APD-, and DRF- values as well as from analysis of presenting symptoms. Patients with no preoperative hydronephrosis (APD < 10 mm) were excluded from analysis of resolution. Patients with reoperation were excluded from analysis of resolution as they did not follow the regular follow-up program.

Surgery was performed as an Anderson-Hynes dismembered pyeloplasty on all patients. A double J stent was routinely inserted during surgery and kept for four weeks. Flank incision, lumbar incision, or robotic approach was used. Robotic assisted surgery was performed when available and if patients were ≥ 15–20 kg. Postoperative ultrasound was performed at 1, 3, 6, 12, and 24 months after the pyeloplasty. MAG3 scans were performed at 3 and 12 months post pyeloplasty.

### Outcomes

Several parameters in the pre, peri- and postoperative course were compared between boys and girls. In the Kaplan–Meier curves, the primary outcome was resolution of hydronephrosis. Resolution was defined as APD < 10 mm [11] or a > 50% decrease in APD compared to preoperative values. When a patient had hydronephrosis preoperatively but no stated APD in the preoperative ultrasound report, a resolution was defined as APD < 10 mm postoperatively. Time to resolution was defined as the first time after surgery that the patient achieved lasting resolution. Postoperative MAG3 values were detected and presented as improved, deteriorated, or stable renal function. A change was considered significant if the change was more than ± 3 percent points. Reoperation was any postoperative intervention with the intention to widen the ureteropelvic junction, for example, balloon dilatation or a new pyeloplasty.

### Independent variables

Independent variables were sex (girls or boys), age at presentation (months), age of surgery (months), time from presentation to surgery (months), presenting symptoms, anterior posterior diameter measures (APD) before surgery (mm), preoperative DRF on MAG3 scan (%), type of surgery and type of obstruction. Age at presentation was defined as when the hydronephrosis or the obstruction was found on imaging. The time from discovery to surgery was calculated by subtracting the age at discovery from the age at surgery. APD measures were collected from the report from the latest ultrasound before surgery. When the patient had a preoperative nephrostomy, the latest value before nephrostomy insertion was used. DRF was collected from the MAG3 scan. Presentation was divided into flank pain, infection (pyelonephritis, urosepsis, or febrile urinary tract infection), no symptoms, and accidentally discovered hydronephrosis.

Surgical approach and type of obstruction were collected from the operating report. Type of obstruction was sorted into intrinsic, extrinsic, or both. Extrinsic obstruction was defined as crossing vessels described in the operative report.

### Statistical analysis

All statistical analyses were performed in SPSS version 28.0. Continuous data were not normally distributed and therefore presented as median (min–max). Categorical data were presented as the absolute number and percentage of patients *n* (%). Descriptive statistics on patient characteristics were produced. Chi^2^ test or Fisher's exact test was used to compare categorical data and the Mann–Whitney *U*-test was used to compare continuous data between sexes. Logistic regression was used to adjust certain analyses for possible confounders, and presented as odds ratio (OR) with 95% confidence intervals (95%CI). Kaplan–Meier curves were produced to chart resolution and differences between sexes were assessed with the log rank test. The significance level was set at *p* < 0.05.

## Results

### Study population

**Fig. 1:**
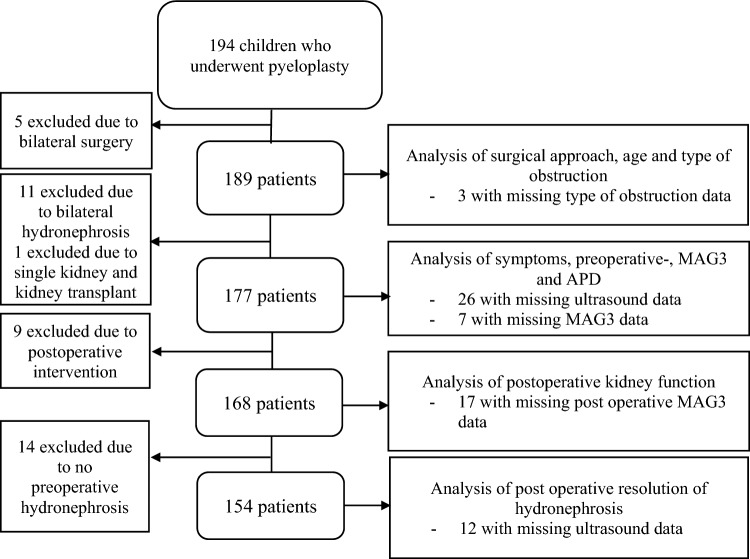
Flowchart of exclusion and inclusion of children who underwent pyeloplasty for ureteropelvic junction obstruction. *MAG3* Tc99m mercaptoacetyltriglycine renography, *APD* anterior posterior diameter

A total of 194 children were operated on with pyeloplasty during the study period. Out of these, 126 (64.9%) were boys. Five patients were excluded from all variables except gender and side of surgery due to bilateral surgery. Eleven patients had bilateral hydronephrosis and were therefore excluded from analysis of symptoms, MAG3, and APD. One patient was excluded because of single kidney and kidney-transplant post pyeloplasty. Analysis of the primary outcome was performed on 142 patients (Fig. 1).

Age at presentation varied widely, the most common symptom was pain, and most children had an internal obstruction. Almost half of the children had a flank incision. Overall, 4.8% needed a re-intervention (Table 1).

**Table 1:** Overview of patient characteristics of 194 children who underwent pyeloplasty for ureteropelvic junction obstruction

	*N* = 194
Gender	
Boys	126 (64.9%)
Girls	68 (35.1%)
Age at discovery (years)	1.0 (0.0–15.7)
Age at surgery (years)	5.0 (0.2–16.7)
Symptoms	
Infection	19 (10.7%)
Pain and infection	18 (10.1%)
Pain	70 (39.3%)
No symptoms	61 (34.3%)
Accidentally found	10 (5.6%)
Preoperative MAG3 (%)	41 (9–61)
Preoperative APD (mm)	30 (5–90)
Side of surgery	
Right	71 (36.6%)
Left	118 (60.8%)
Bilateral	5 (2.6%)
Surgical approach	
Flank incision	100 (52.9%)
Lumbotomy	30 (15.9%)
Robotic assisted	59 (31.2%)
Type of obstruction	
Internal	127 68.3%)
External	53 (28.5%)
Both internal and external	6 (3.2%)
Postoperative intervention	
Yes	9 (4.8%)
No	179 (95.2%)

Presented as absolute number (percentage of patients) or median (minimum–maximum)

Five patients were excluded from all variables except gender and side of surgery due to bilateral surgery. Eleven patients with bilateral hydronephrosis were excluded from symptoms, MAG3, and APD. One patient was excluded because of single kidney and kidney-transplant post pyeloplasty. Reduced data due to missing values in preoperative MAG3 (*N* = 171), preoperative APD (*N* = 152), and type of obstruction (*N* = 186)

*MAG3* Tc99m mercaptoacetyltriglycine diuretic renography, *APD*—anterior posterior diameter

### Presentation

No significant differences were found between the sexes in rate of prenatal hydronephrosis, or in preoperative APD or DRF. There was also no difference in when the UPJO was discovered or in the time between discovery and surgery. A higher rate of boys had pain as presenting symptom (47.4% vs. 25.0%, *p* < 0.01) while more of the girls presented with infection (17.2% vs. 7.0%, *p* = 0.04). It was also more common for girls to have a combination of pain and infection (*p* = 0.02). Almost 11% of the girls had accidentally found hydronephrosis compared to 2.6% of boys (*p* = 0.04). Five boys were operated bilaterally compared to zero girls (4% to 0%, *p* = 0.164) (Table 2). When adjusting for age, preoperative anterior/posterior-measurement and differential renal function on MAG3, and type of obstruction; the risk of experiencing pain only as the presenting symptom was four times higher in boys compared to girls (adjusted OR 4.3 [95% CI 1.8–10.4]).

**Table 2:** Comparison of preoperative factors between boys and girls who underwent pyeloplasty for ureteropelvic junction obstruction

	Boys (*N* = 121)	Girls (*N* = 68)	*p* value
Age			
At discovery (years)	1.3 (0–15.7)	0.5 (0–14.8)	0.668^a^
At surgery (years)	5.4 (0.2–16.7)	3.7 (0.5–15.1)	0.740^a^
Time, discovery to surgery (months)	9 (0–160)	11 (0–144)	0.482^a^
Prenatal hydronephrosis	57 (47.1%)	32 (47.1%)	0.995^b^
Symptoms			
Infection	8 (7.0%)	11 (17.2%)	0.044^b^
Pain and infection	7 (6.1%)	11 (17.2%)	0.023^b^
Pain	54 (47.4%)	16 (25.0%)	0.004^b^
No symptoms	42 (36.8%)	19 (29.7%)	0.441^b^
Accidentally found	3 (2.6%)	7 (10.9%)	0.037^c^
Side			0.649^b^
Right	44 (36.4%)	27 (39.7%)	
Left	77 (63.6%)	41 (60.3%)	
Preoperative APD (mm)	30 (5–70)	30 (6–90)	0.806^a^
Preoperative MAG3 (%)	41 (11–57)	38.5 (9–61)	0.402^a^

Presented as absolute number (percentage of patients) and median (minimum–maximum)

Eleven patients with bilateral hydronephrosis were excluded from symptoms, MAG3, and APD. One patient was excluded because of single kidney and kidney-transplant post pyeloplasty. Reduced data due to missing values in preoperative MAG3 (*N* = 171), preoperative APD (*N* = 152) and type of obstruction (*N* = 186)

*MAG3* Tc99m mercaptoacetyltriglycine diuretic renography, *APD*—anterior posterior diameter

^a^Mann–Whitney test

^b^Chi-square test

^c^Fishers exact test

### Surgical characteristics

No differences in type of surgical approach could be seen. Overall, 71% of the boys had an internal obstruction compared to 63% of the girls. Out of the total 127 patients who had an internal obstruction, one patient had an intraluminal polyp, and two patients had a ureteric fold (Table 2).

### Postoperative follow-up

**Fig. 2:**
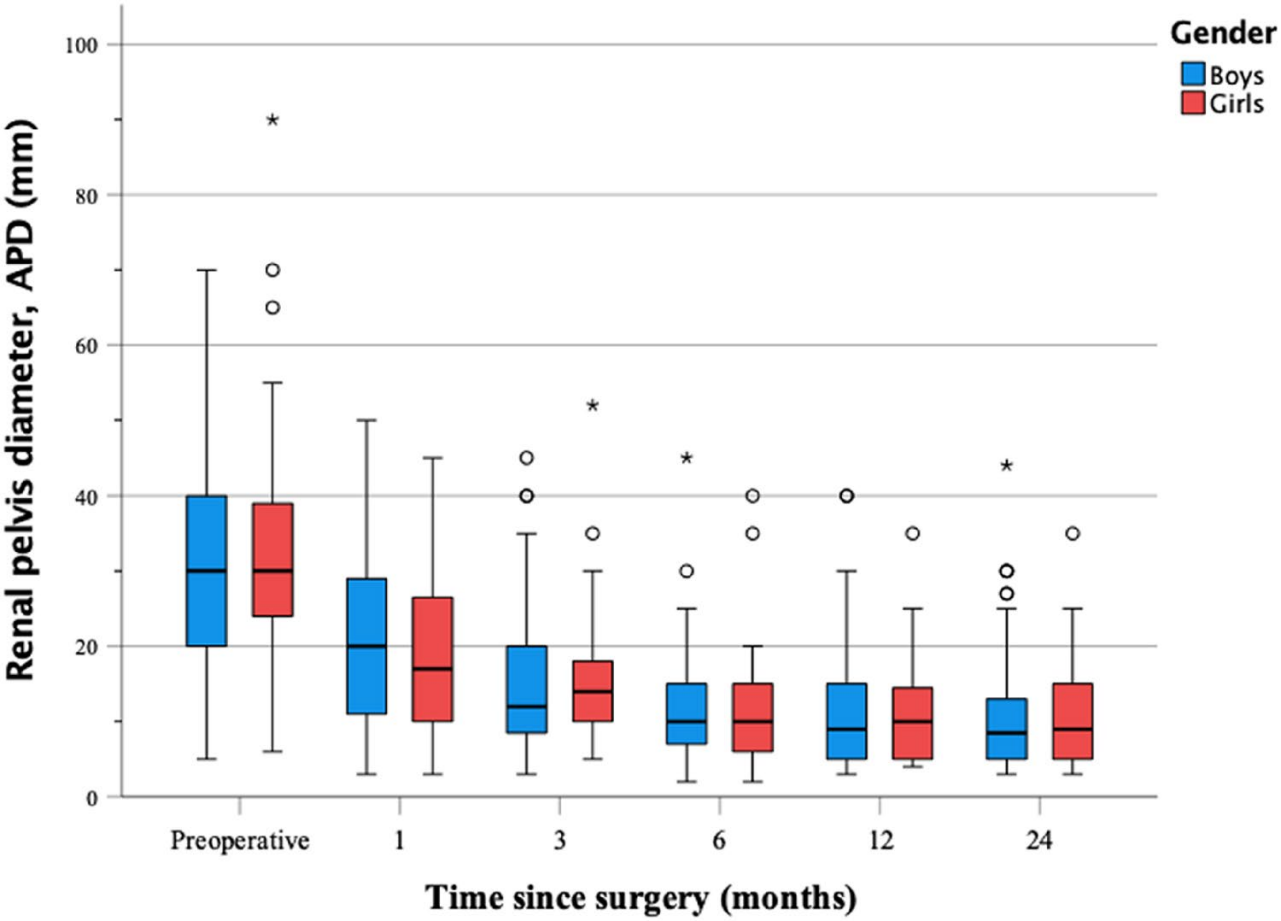
Median renal pelvis diameter APD (anterior posterior diameter) between boys and girls during 24 months follow-up after pyeloplasty for ureteropelvic junction obstruction. *N* = 142

There were no significant differences between sexes in APD on the follow-up US at 1, 3, 6, 12 and 24 months (*p* = 0.231, *p* = 0.619, *p* = 0.603, *p* = 0.674, *p* = 0.856, respectively) (Fig. 2). Almost half (46.4%) achieved an increase in DRF on the operated side postoperatively with no significant difference between sexes. Out of those with unchanged DRF, the median preoperative value was 45% (13–57%). A total of 9% had a decreased DRF, out of these 14 patients, eight (57%) received resolution of hydronephrosis.

**Fig. 3:**
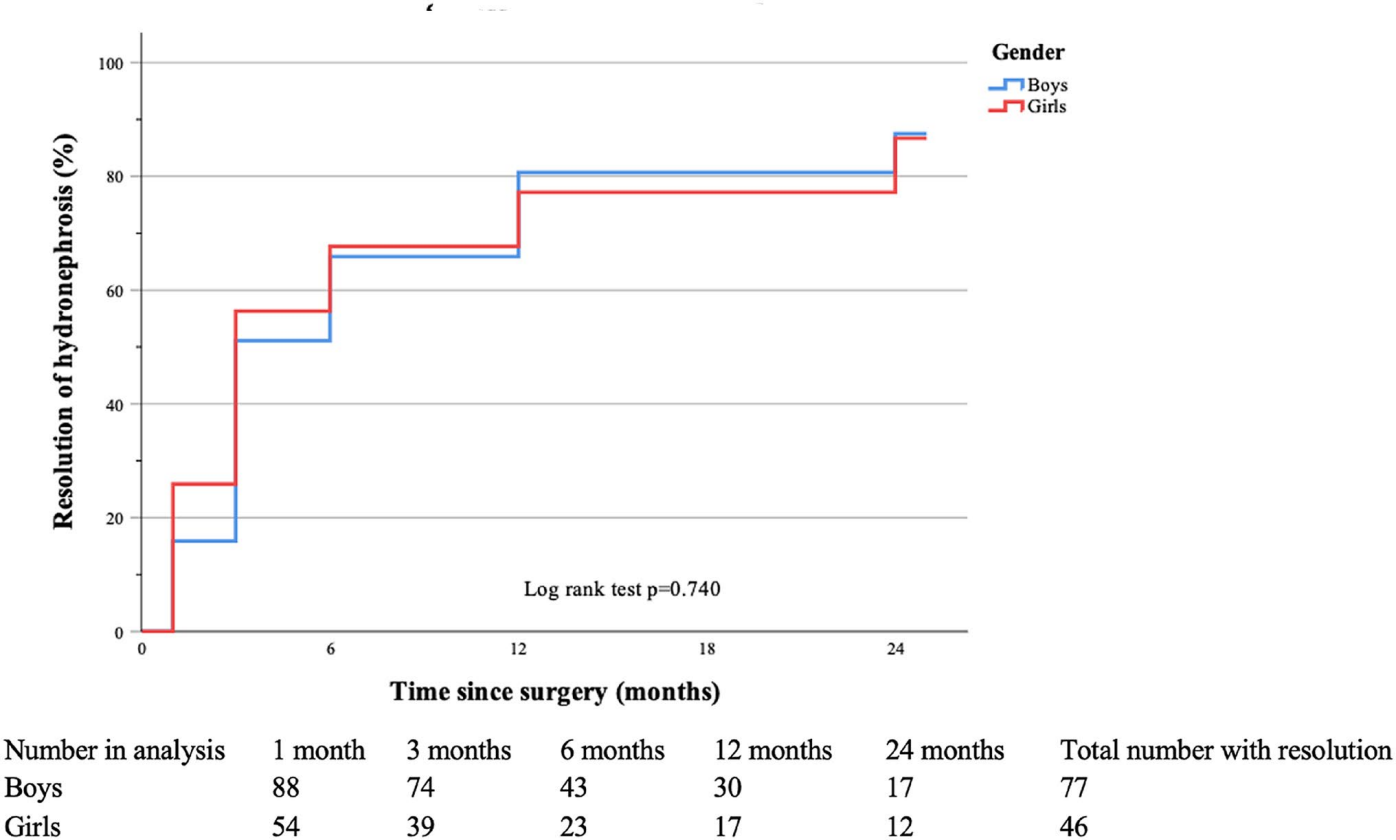
Kaplan–Meier curve of resolution of hydronephrosis after pyeloplasty with comparison between boys and girls

At follow-up, a total of 87.5% of the boys and 85.2% of the girls achieved resolution with no significant difference (*p* = 0.889). There was also no difference in resolution of hydronephrosis between sexes in the Kaplan–Meier curve (log rank *p* = 0.740) (Fig. 3). The ratio of resolution in the whole cohort was 86.6%. Boys and girls achieved resolution at the same time at a median of 3 months (Table 3).

**Table 3:** Comparison of surgical and postoperative factors between boys and girls who underwent pyeloplasty for ureteropelvic junction obstruction

	Boys * N* = 121	Girls * N* = 68	*p* value
Surgical approach			0.383^b^
Flank	61 (50.4%)	39 (57.4%)	
Lumbotomy	18 (14.9%)	12 (17.6%)	
Robotic assisted	42 (34.7%)	17 (25%)	
Kind of obstruction			0.410^c^
Internal	86 (71.1%)	41 (63.1%)	
External	32 (26.4%)	21 (32.3%)	
Both external and internal	3 (2.5%)	3 (4.6%)	
Resolution	77 (87.5%)	46 (85.2%)	0.889^b^
Time to resolution (months)	3 (1–24)	3 (1–24)	0.371^a^
MAG3 change postoperative			0.074^b^
Increased	35 (38.9%)	35 (57.4%)	
Decreased	9 (10.0%)	5 (8.2%)	
Unchanged	46 (51.1%)	21 (34.4%)	
Postoperative intervention	9 (7.5%)	0 (0%)	0.028^c^

Presented as absolute number (percentage of patients) and median (minimum–maximum)

One patient was excluded from MAG3, resolution, and postoperative intervention because of single kidney and kidney-transplant post pyeloplasty. Eleven patients with bilateral hydronephrosis were excluded from MAG3, and resolution. Nine patients were excluded from postoperative resolution and MAG3 due to postoperative intervention. 14 were excluded from the analysis of resolution due to no preoperative hydronephrosis. Reduced data due to missing values in postoperative MAG3 (*N* = 151), resolution (*N* = 142)

*MAG3* Tc99m mercaptoacetyltriglycine renography

^a^Mann–Whitney test

^b^Chi-square test

^c^Fishers exact test

A total of nine (4.8%) children needed a secondary surgery for their PUJO of which all were boys (*p* = 0.028). Two had leakage from the anastomosis and two had urosepsis. Two had recurring symptoms in the form of one patient with pyelonephritis and one with recurring flank pain. Three had remaining obstruction on imaging.

## Discussion

The purpose of this study was to evaluate potential differences between boys and girls who underwent pyeloplasty for UPJO. Because UPJO is, for largely unknown reasons, much more common in boys [3–5], other sex differences in this condition could exist but has never been evaluated or reported. While most of the studied parameters did not differ between girls and boys, differences in presenting symptoms and in the rate of reoperations were found.

It is important to notice delays in diagnosis and surgery. We found no differences in at what age the hydronephrosis was discovered or in the age at the time of surgery between the sexes. One previous study found that boys with PUJO were often operated on earlier [20]. The discrepancy may be due to the American cohort studied with, for example, insurance status, which they also found to affect age at surgery [20]. The time from discovery to surgery may not be equivalent to waiting time as it may take years from discovery to surgery indication. Nonetheless, a difference would have indicated either being more prone to operate or PUJO having a more severe course in either of the sexes. It could also point to different types of obstruction between the sexes since internal obstruction often presents prenatally and hence leads to earlier surgery. Coherently, discovering the hydronephrosis prenatally was also equally common in both genders in the present study. This result is consistent with Nordenström et al. [10].

The operating technique was determined by several aspects. One important factor may be the weight of the child which is strongly affected by age [20, 21]. There was no difference between sexes in operating techniques in our cohort, however, not adjusted for age. Other aspects than time to surgery that may indicate a more severe course of the disease are preoperative APD and DRF. In both, we found no significant differences. If for example, boys would have worse preoperative kidney function but the same waiting time, that would indicate the need for more active management of boys. Fortunately, this was not the case in our cohort as both timing and preoperative imaging variables seem to be equal.

Knowing the symptomology of a disease is important and it may alter between sexes. For example, women are more likely to report atypical symptoms of coronary heart disease than men which may lead to diagnostic challenges [22]. The current study found a significant difference in the symptomatology, with girls seeming more prone to suffer from infection while boys were more prone to experience pain only. Accidentally found hydronephrosis and the combination of pain and infection was also more common among girls. Urinary tract infections are generally more common for girls above the age of 6 months [23, 24]. In Sweden, children with a febrile UTI should routinely be evaluated with a renal ultrasound according to the Swedish Pediatric Society Guidelines [25]. It is possible that the higher ratio of infection in the current study reflects the general higher incidence of UTI among girls, especially due to vesicoureteral reflux (VUR), and that hydronephrosis was discovered accidentally in connection with the infection. Hence, most children included had a voiding cystourethrogram in their preoperative work-up, but some of the children (especially girls) with infection as a presenting symptom may have had a low-grade VUR. However, it requires further investigation. Nonetheless, PUJO should be considered for children in general and girls in particular with febrile UTIs. A higher percentage of boys experienced flank pain as presenting symptoms. One possibility is perhaps that girls for sociocultural reasons do not express pain to the same extent. Another reason might be biological or anatomical reasons. Cain et al. found it was more common to present with pain when one has extrinsic obstruction [6]. Contradictory to our results, a previous study found a higher incidence of crossing vessels among girls [19]. Differences in study methods may be the reason for this. For example, Menon et al. had different indications for surgery, not based on DRF [19]. This could result in slightly different characteristics in the study population. Anyhow, crossing vessels do not seem to be the reason for a higher ratio of pain among boys in our cohort. Further research should explore the mechanisms behind the differences in symptomology.

We further established that pyeloplasty leads to resolution of hydronephrosis in a majority of patients and also found no difference between the sexes. However, it is important to notice that a successful surgery also includes improvement of symptoms and improved DRF or drainage on MAG3 scan. A total of 4.8% in this study required a reoperation. This seems to correspond with previous studies with rates of failed pyeloplasty at 3.1–5.9% [26, 27]. However, the present study found a significantly higher percentage of boys who needed additional intervention, 7.5% compared to 0% girls. There is very limited research on the sex distribution of postoperative interventions on pyeloplasty. Dy et al. analyzed a large cohort of children who underwent pyeloplasty and found no differences in which sex needed reoperation [28]. However, the definition of postoperative interventions was different as Dy et al. also included postoperative stents. It is hard to say what differences in outcome between sexes derive from [10–12]. Sociocultural reasons may be one explanation. It is a possibility that boys do not express their early signs of complications or are not as vigilantly tested as girls. For example, Zachariasse et al. found girls received more diagnostic testing at the emergency department, although the cause for this is also unknown [17]. There may also be biological reasons. It is known that female trauma patients have better outcomes in trauma care, hypothetical caused by women´s higher estrogen levels [29, 30]. How this is transferable to the trauma of surgery and to a pediatric population is unsure. Further research is needed to evaluate the reasons for boys’ poorer surgical outcomes.

A strength of this study is that it includes all patients being operated with pyeloplasty at a tertiary center for between the years of 2002–2020. However, a retrospective study design dispenses several difficulties. Imaging techniques and measurements have developed during the almost 20 years this study comprises which could create imprecise variations. Also, there may be variations in how radiologists term renopelvic dilation [5]. Additionally, the interpretation of medical records is sometimes a challenge. It is dependent on the interpretation of individual physicians' formulations, which sometimes are vaguely formulated. The missing data during follow-ups may lead to bias. Not all analyzes were adjusted for potential confounders and we also did not adjust for multiple analyses. These factors could lead to overestimation of the significance of sex in our results. On the other hand, it is hard to explain why any of the known weaknesses in data collection in retrospective studies, should be more present with regard to boys or girls.

## Conclusion

In summary, girls seem to experience infections as presenting symptoms for PUJO more often than boys, while boys significantly more often present with pain. PUJO is discovered and operated at the same time and has equal preoperative findings on imaging in both sexes. Surgical characteristics and postoperative resolution and improvement are generally the same after pyeloplasty, but a higher rate of boys seem to need reoperation. This information is valuable in future studies on outcome after pyeloplasty and as information to physicians handling children with PUJO. However, further research will be necessary to investigate the mechanisms behind sex differences in pelvoureteric junction obstruction.

## Data Availability

The data that support the findings of this study are available from the corresponding author upon reasonable request.
